# The Skin Microbiome of the Neotropical Frog *Craugastor fitzingeri*: Inferring Potential Bacterial-Host-Pathogen Interactions From Metagenomic Data

**DOI:** 10.3389/fmicb.2018.00466

**Published:** 2018-03-20

**Authors:** Eria A. Rebollar, Ana Gutiérrez-Preciado, Cecilia Noecker, Alexander Eng, Myra C. Hughey, Daniel Medina, Jenifer B. Walke, Elhanan Borenstein, Roderick V. Jensen, Lisa K. Belden, Reid N. Harris

**Affiliations:** ^1^Department of Biology, James Madison University, Harrisonburg, VA, United States; ^2^Unité d'Ecologie, Systématique et Evolution, Université Paris-Sud, Paris, France; ^3^Department of Genome Sciences, University of Washington, Seattle, WA, United States; ^4^Department of Biological Sciences, Virginia Tech, Blacksburg, VA, United States; ^5^Department of Computer Science and Engineering, University of Washington, Seattle, WA, United States; ^6^Santa Fe Institute, Santa Fe, NM, United States; ^7^Smithsonian Tropical Research Institution, Panama City, Panama; ^8^Amphibian Survival Alliance, London, United Kingdom

**Keywords:** skin microbiome, shotgun metagenomics, host-bacteria interactions, amphibians, *Batrachochytrium dendrobatidis*

## Abstract

Skin symbiotic bacteria on amphibians can play a role in protecting their host against pathogens. Chytridiomycosis, the disease caused by *Batrachochytrium dendrobatidis*, Bd, has caused dramatic population declines and extinctions of amphibians worldwide. Anti-Bd bacteria from amphibian skin have been cultured, and skin bacterial communities have been described through 16S rRNA gene amplicon sequencing. Here, we present a shotgun metagenomic analysis of skin bacterial communities from a Neotropical frog, *Craugastor fitzingeri*. We sequenced the metagenome of six frogs from two different sites in Panamá: three frogs from Soberanía (Sob), a Bd-endemic site, and three frogs from Serranía del Sapo (Sapo), a Bd-naïve site. We described the taxonomic composition of skin microbiomes and found that *Pseudomonas* was a major component of these communities. We also identified that Sob communities were enriched in Actinobacteria while Sapo communities were enriched in Gammaproteobacteria. We described gene abundances within the main functional classes and found genes enriched either in Sapo or Sob. We then focused our study on five functional classes of genes: biosynthesis of secondary metabolites, metabolism of terpenoids and polyketides, membrane transport, cellular communication and antimicrobial drug resistance. These gene classes are potentially involved in bacterial communication, bacterial-host and bacterial-pathogen interactions among other functions. We found that *C. fitzingeri* metagenomes have a wide array of genes that code for secondary metabolites, including antibiotics and bacterial toxins, which may be involved in bacterial communication, but could also have a defensive role against pathogens. Several genes involved in bacterial communication and bacterial-host interactions, such as biofilm formation and bacterial secretion systems were found. We identified specific genes and pathways enriched at the different sites and determined that gene co-occurrence networks differed between sites. Our results suggest that skin microbiomes are composed of distinct bacterial taxa with a wide range of metabolic capabilities involved in bacterial defense and communication. Differences in taxonomic composition and pathway enrichments suggest that skin microbiomes from different sites have unique functional properties. This study strongly supports the need for shotgun metagenomic analyses to describe the functional capacities of skin microbiomes and to tease apart their role in host defense against pathogens.

## Introduction

Microbial symbiotic communities are ubiquitous in animals and plants. For decades, most of the studies in animals have focused on insect endosymbionts, which generally involve only a few bacterial species (Dale and Moran, 2006), whereas more recent studies, mainly in humans, have revealed a complex community of microbes coexisting with their host (Turnbaugh et al., 2007; Grice, 2014; Jorth et al., 2014). Many of these symbionts complete a variety of functions for their hosts, such as nutrient acquisition (Dale and Moran, 2006) and pathogen protection (Fraune et al., 2014; Walke and Belden, 2016). Recent studies have determined that the skin of amphibians harbors bacterial communities that are unique to different species (McKenzie et al., 2012; Kueneman et al., 2014; Walke et al., 2014; Belden et al., 2015; Costa et al., 2016; Rebollar et al., 2016; Bletz et al., 2017a) and might play an important role in protecting the host against *Batrachochytrium dendrobatidis*, Bd (Harris et al., 2009; Becker et al., 2011; Kueneman et al., 2016). This pathogenic fungus causes a skin disease called chytridiomycosis, and it has been considered one of the greatest global threats to amphibian populations (Fisher et al., 2009; Kilpatrick et al., 2010). Field studies have shown that not all species are susceptible to Bd, as some species persist in Bd-endemic regions with no apparent population declines (Crawford et al., 2010; Rebollar et al., 2014; Rodríguez-Brenes et al., 2016). One important component of defense against pathogens that could explain the presence of tolerant and resistant frog species is skin symbiotic bacteria. Hundreds of Bd-inhibitory bacteria have been isolated from the skin of multiple amphibian species from many sites around the world (Antifungal Isolates Database: Woodhams et al., 2015). Moreover, the addition of some of these bacteria (e.g., *Janthinobacterium lividum*) to captive amphibians protected the hosts from Bd infection and reduced morbidity and mortality (Harris et al., 2009; Becker et al., 2011; Kueneman et al., 2016). In addition, skin bacterial community structure before infection can predict mortality and morbidity after infection (Becker et al., 2015a; Walke et al., 2015).

Even though our knowledge of amphibian skin microbial communities has grown considerably in the past decade, we still lack knowledge of the full range of functional capabilities of these communities and their interactions with their hosts. Most of the studies have focused on describing the skin community via 16S rRNA gene amplicon sequencing (Kueneman et al., 2014; Rebollar et al., 2016; Bletz et al., 2017a) and/or culturing bacteria to characterize their inhibitory capacities against Bd using *in vitro* challenge assays (Harris et al., 2006; Flechas et al., 2012; Bell et al., 2013; Becker et al., 2015b; Medina et al., 2017). In other cases, functions have been predicted using bacterial data bases (Kueneman et al., 2015; Bletz et al., 2017b) or predictive tools such as PICRUSt (Loudon et al., 2014a). Moreover, changes in skin bacterial community structure associated with Bd infection have been observed in experimental settings (Jani and Briggs, 2014; Becker et al., 2015a; Walke et al., 2015; Longo and Zamudio, 2017); however, we still lack knowledge of the functional changes that may occur in the bacterial community once Bd is present.

Recent studies on several symbiotic systems have identified genes that were originally described in pathogens, which are also important in mutualistic bacterial species, such as bacterial secretion systems (Dale and Moran, 2006; Preston, 2007; Medina and Sachs, 2010) and biofilm formation (Frese et al., 2013; Schmid et al., 2015). In addition, the host can also affect the symbiotic community through the production of molecules, such as antimicrobial peptides (AMPs) (Gallo and Hooper, 2012; Franzenburg et al., 2013). Furthermore, the study of bacterial symbionts on other animal hosts, like amphibians, has revealed symbiotic bacterial interactions that are important in protecting the hosts against pathogens (Walke and Belden, 2016). One mechanism through which bacteria protect their host is through the production of secondary metabolites that have antimicrobial properties (Flórez et al., 2015). In amphibians, some members of the skin microbiota can produce secondary metabolites that inhibit the growth of the fungal pathogen Bd. Examples of these antifungal metabolites are violacein (Brucker et al., 2008b), prodigiosin (Woodhams et al., 2017), tryptophol (Loudon et al., 2014b), indole-3-carboxaldehyde (I3C) (Brucker et al., 2008b) and 2,4-diacetylphloroglucinol (2,4 DAPG) (Brucker et al., 2008a).

Here, we describe the genes involved in bacterial defense and communication that are present in skin microbiomes of the terrestrial Neotropical frog *Craugastor fitzingeri* using shotgun metagenomics. Our *a priori* goals were to expand our knowledge of the metabolic capacities that symbiotic bacteria have and to explore the presence of genes that are potentially involved in bacteria-bacteria, bacteria-host and bacteria-pathogen interactions. Previous studies have described the skin bacterial structure of *C. fitzingeri* across several regions in Panamá (Belden et al., 2015; Rebollar et al., 2016). These studies showed that the skin bacterial community is dominated by the phyla Proteobacteria and Actinobacteria. Specifically, Rebollar et al. (2016) described differences in bacterial OTU relative abundance in frog skin communities between a Bd-endemic site and a Bd-naïve site. The bacterial community structure in the Bd-endemic site was enriched for taxa known to have antifungal properties (e.g., *Pseudomonas* and members of the Actinobacteria class). These differences may be related to natural selection caused by the presence or absence of Bd in these lowland regions, although other explanations are possible.

In this study, we analyzed samples from three individuals of *C. fitzingeri* from a Bd-endemic site and three from a Bd-naïve site. These samples were previously analyzed by Rebollar et al. (2016) with 16S rRNA gene amplicon sequencing for bacterial community profiling. We described the taxonomic composition and analyzed the genetic metabolic pathways present in skin microbiomes. Lastly, we compared the bacterial composition and function among frogs from a Bd-endemic site and a Bd-naïve site. We hypothesized that frog skin microbiomes will include genes associated with bacterial communication and bacterial-host interactions, as well as pathways involved in the production of antifungal metabolites and resistance to bacterial toxins, as a result of potential cooperative or competitive interactions within the community. We also hypothesized that taxonomic and functional composition of skin microbiomes will differ between sites with contrasting Bd incidence. A finding of genes associated with bacterial-host interactions would increase our confidence that amphibian skin bacteria are resident species and not transient. Moreover, unraveling the potential functions present in these bacterial communities will advance our knowledge of the interactions occurring among the host, the bacterial symbiotic community and the pathogen, Bd.

## Methods

### Sample selection and molecular procedures

*C. fitzingeri* frogs were collected and swabbed from two lowland forests sites in Panamá: Soberanía National Park (Sob), a Bd endemic site, and Serranía del Sapo (Sapo), a Bd naïve site. DNA from these swab samples was extracted and used in a previous study for 16S amplicon sequencing and qPCR detection of Bd (Rebollar et al., 2016), which contains the details of the sample collection and the molecular procedures used to extract the DNA from the swabs. Six DNA samples extracted from this previous study were used to sequence the metagenome as explained in section Shotgun metagenome sequencing. We chose these samples considering their Bd infection status (infected vs. not infected) and collection site (Bd endemic and Bd naïve) (Table S1). Two other samples were sequenced but were not included in further analyses because they could not be properly annotated.

### Shotgun metagenome sequencing

Six DNA samples (three from Sob and three from Sapo) were used to obtain frog skin shotgun metagenomes (Table S1). The six barcoded samples were randomly distributed on two lanes (three samples on each lane) and were sequenced on HiSeq 2500 (Genomics Sequencing Center, Bioinformatics Institute, Virginia Tech), generating over 500 million, 100 bp, paired end reads (with ~200 bp insert size). The numbers of reads for each of the six samples ranged from 84 million to 124 million reads (Table S1). Metagenomic reads were deposited in the SRA Database (NCBI) with the accession number SRP130893 as part of Bioproject PRJNA429199.

### Metagenome binning for filtering out host reads

With the purpose of filtering out the host (*C. fitzingeri*) reads, we assembled the reads for each of the six samples into metagenome contigs using Ray Meta (Boisvert et al., 2012) *de novo* assembler. Four million reads for each frog sample were then mapped to all the contigs greater than 1,000 bp from all six frogs to determine the relative abundance of each contig. The covariance of these abundances across samples was then used to cluster all contigs with >100 mapped reads into metagenome bins using the PAM k-medoids algorithm in R with *k* = 10. Contigs in three of these bins or clusters (Clusters 1–3) exhibited similar abundance profiles across the six samples and appeared to be predominantly frog (with consistently low GC content ~42% and best Blastn hits to eukaryotic sequences). We also mapped the reads for each sample to the frog 18S rRNA sequence (assembled from the data) and a database of bacterial 16S rRNA sequences (from www.patricbrc.org) to estimate the relative proportion of eukaryotic (mainly frog) and prokaryotic (mainly bacteria) DNA from each sample. The 18S/16S ratios for the six samples showed good correspondence with the variation in the abundance of the frog DNA clusters. The remaining seven clusters (Cluster 4–10) were composed of contigs belonging to different bacterial groups.

### Taxonomic assignment and procrustes analysis

We determined the bacterial species composition of the frog skin samples using Metaphlan (Segata et al., 2012), a method used to profile bacterial communities based on clade-specific marker genes. We determined the relative abundance of bacterial taxa at different taxonomic levels based on the annotated reads that had been previously filtered to eliminate host-derived reads (Table S1).

We used a Procrustes analysis to evaluate how similar the 16S rRNA gene amplicon sequencing data (obtained with QIIME in Rebollar et al., 2016) was to the shotgun metagenome data (obtained with Metaphlan) using the vegan R package (R Core Team, 2015; Oksanen et al., 2016). Procrustes analysis evaluates the congruency between two data sets by the superimposition of principal component analyses (McHardy et al., 2013; Luo et al., 2016). We performed Procrustes analysis on Bray-Curtis distance matrices calculated from the bacterial relative abundance at the genus level in both data sets. We used the PROTEST test function from the vegan R package (R Core Team, 2015; Oksanen et al., 2016), which performs repeated symmetric Procrustes analyses to estimate if the degree of concordance between matrices is greater than expected by random association (Jackson, 1995). Significant *p*-values below 0.05 indicate that the matrices are more similar than expected by random association.

### Read level functional annotation

To annotate the metagenome reads (read level analysis), we assigned reads to the bacterial clusters obtained in section Metagenome binning for filtering out host reads by re-mapping reads from each sample to contigs assembled from the same sample using blastn (Altschul et al., 1990) with an e-value cutoff of 1. This resulted in 8,323,746 reads (1.57% out of the total number of reads) that mapped to contigs with frog-associated cluster assignments, which were discarded.

We assigned KEGG orthology (KO) group (Kanehisa and Goto, 2000) annotations to non-frog-associated reads from the six frog samples using blastx with an e-value cutoff of 1 to map reads to the KEGG gene database downloaded on July 15th, 2013. This produced 192,761,569 (36.36%) reads with best hits to genes with KO annotations (Table S1). We calculated KO abundances using the number of reads assigned to each KO. In the case where a read had best hits to multiple KOs, the read count was evenly distributed among the corresponding KOs. For example, if a read mapped equally well to 3 KOs, each KO received 1/3 of the count. We normalized the abundances of KOs using MUSiCC (Manor and Borenstein, 2015) with the inter-sample correction option, which corrects for biases in quantification using the abundance of universal single-copy KOs.

### Descriptive and comparative analyses using read level annotations

To evaluate differences in the functional repertoire among frog skin communities at multiple levels of detail, we aggregated the annotated KO assignments into modules, pathways, and classes based on a custom-curated version of the BRITE hierarchy. We summed the relative abundances of all KOs associated with each pathway, module, or class, following Manor et al. (2016). Pathways and modules were further filtered to verify that downstream analysis considers only bacterial pathways or modules. Specifically, a pathway or module was included in our analysis only if at least 1% of bacterial genomes in KEGG contained at least 1 KO from that pathway or module, and if these bacterial genomes contained at least 5% (20%) of the KOs in the pathway or module, on average.

For each pathway, module or class, normalized abundances from samples from the Sapo region were compared to those from Sob using LefSe Analysis (Linear Discriminant Analysis Effect Size) which incorporates the use of two non-parametric tests (Kruskal-Wallis and Wilcoxon) and a linear discriminant analysis to detect biomarkers or genes that are differentially abundant among the groups tested (Segata et al., 2011).

### Contig level functional annotation and assembly

To determine the taxonomic identity of the genes present on frog skin metagenomes we assembled reads into contigs (contig level analysis), annotated them and determined their taxonomy (Table 1). Reads were assembled into contigs using stringent criteria to facilitate gene prediction. Forward and reverse reads were assembled using MEGAHIT version 1.3.0 (Li et al., 2016) with default parameters, except for a minimum length of 200 bp for the assembled contigs and a starting kmer size of 23 in increasing steps of 10 until reaching a kmer size of 93. Gene prediction was performed on the newly assembled contigs using Prokka (Seemann, 2014). For metabolic and taxonomic classifications of the predicted genes, we used GhostKOALA from KEGG (Kanehisa et al., 2016).

**Table 1:** Assembly and annotation data of the six skin metagenomes of *C. fitzingeri* from sites Sapo (*N* = 3) and Sob (*N* = 3).

**Sample ID**	**Assembly**				**Annotation**			
	**Contigs**	**Max(bp)**	**avg (bp)**	**N50**	**CDS**	**miscRNAs**	**tRNAs**	**rRNAs**
Sapo01	311185	154616	501	458	79219	759	704	205
Sapo02	187876	404914	603	568	75067	885	877	134
Sapo03	400815	278576	655	702	231429	2431	3324	195
Sob01	407419	232836	561	516	139736	1196	1548	218
Sob02	148664	42792	703	874	93277	868	1025	66
Sob03	99132	250402	921	1532	82916	858	1379	80

### Descriptive and comparative analyses using contig level annotations

Metabolic and taxonomic data inferred from GhostKOALA were visualized in stacked bar charts using *ad hoc* scripts in R (R Core Team, 2015). We plotted gene counts from five broad functional classes based on KEGG classification: Membrane Transport (MT), Cellular Communication (CC), Metabolism of Terpenoids and Polyketides (MTP), Biosynthesis of Secondary Metabolites (BSM) and Antimicrobial Drug Resistance (ADR). Functional classes or pathways with abundance values < 3 were not plotted. We used LefSe analysis to test for differentially enriched genes (KOs) between sites for all five broad classes and for all the pathways contained within each class (55 pathways in total). Methods on co-occurrence network construction can be found on Supplementary Methods.

## Results

### Bacterial composition of *C. fitzingeri* skin microbiomes

**Figure 1:**
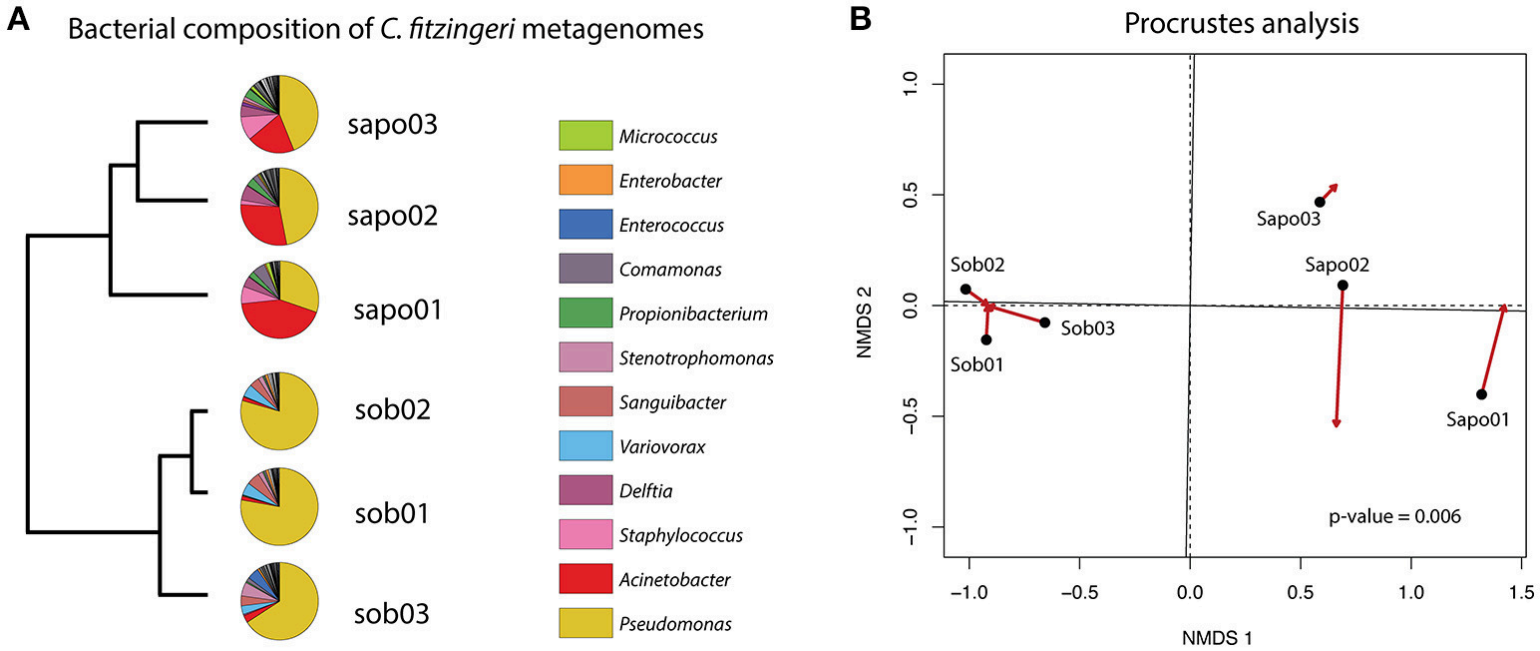
**(A)** Pie charts of the most abundant genera obtained with metaphlan for each frog microbiome sample. An UPGMA on the left shows grouping of similar samples based on the relative abundance of bacterial taxa. **(B)** Procrustes analysis comparing the relative abundance of bacterial taxa (Bray-Curtis dissimilarity matrices) of the 16S rRNA gene amplicon data (arrowheads) and the shotgun metagenome data (black circles). A *p*-value of 0.006 (PROTEST test) indicates that the matrices are more similar than expected by random association.

We determined the taxonomic composition and relative abundance of the bacteria present in the frog metagenomes from *C. fitzingeri*. Based on a Metaphlan analysis of the skin metagenome reads, the dominant genera in the Bd-endemic site (Sob) were *Pseudomonas* (74.43%), *Variovorax* (4.72%), *Sanguibacter* (4.65%) and *Stenotrophomonas* (3.23%), while the Bd-naïve site (Sapo) was dominated by *Pseudomonas* (40.46%), *Acinetobacter* (30.6%), *Staphylococcus* (6.38%) and *Delftia* (5.15%). A hierarchical clustering analysis based on the relative abundance of bacterial taxa suggested that the community structure was different between the three samples from Sob and the three samples from Sapo (Figure 1A). To determine whether the taxonomic composition obtained with 16S rRNA gene amplicon sequencing (Rebollar et al., 2016) was congruent with the shotgun metagenome approach presented in this study, we performed a Procrustes analysis (Figure 1B). Our results indicate that both methods gave similar results when trying to define the dominant groups present on amphibian skin bacterial communities (PROTEST nperm = 999 *p* = 0.0069).

### Presence of main functional classes and gene relative abundance in *C. fitzingeri* skin microbiomes

**Figure 2:**
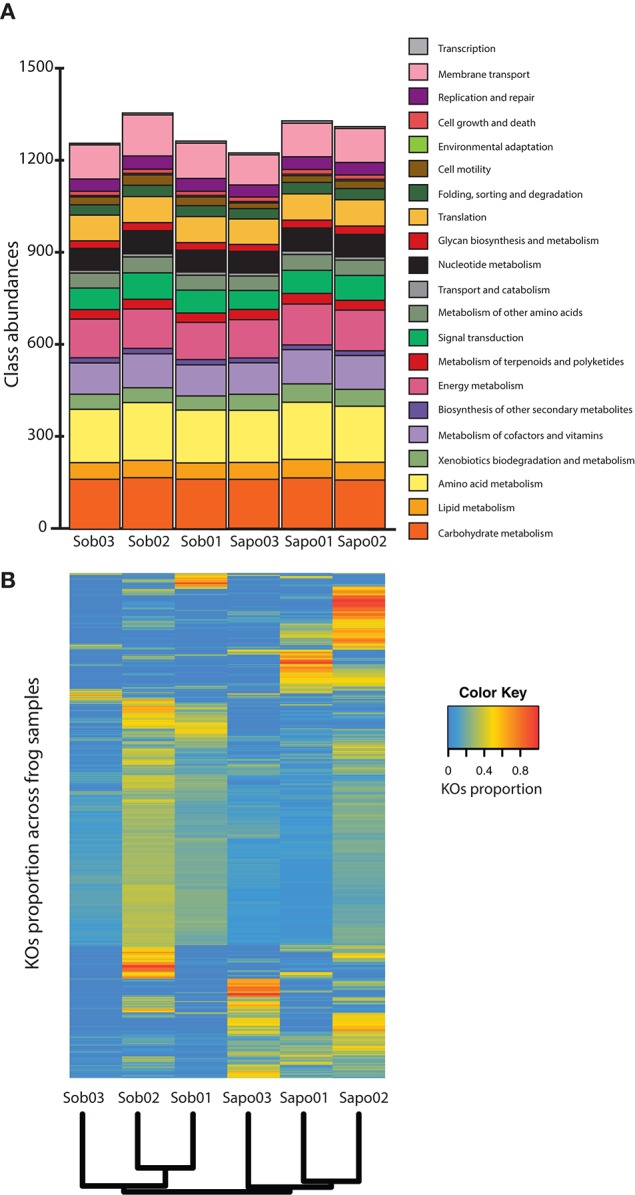
Read level analysis **(A)** Class abundances normalized with MUSiCC. **(B)** Heatmap showing the relative abundance of KOs across samples. Rows are individual KOs and columns are frog samples. Dendogram at the bottom indicates clustering of samples based on Bray-Curtis distances. Colors indicate the relative abundance (proportions) of KOs across samples (see color legend on the right hand side of the figure).

We annotated between 25 and 47% of the reads from the six samples obtained from *C. fitzingeri* skin (Table S1) and classified them according to main functional classes from KEGG. Skin microbiomes contained most of the functional classes identified in KEGG and had a similar proportion of classes among samples (Figure 2A). The most abundant gene classes were amino acid metabolism (mean = 13.87%, SD = 0.11), carbohydrate metabolism (mean = 12.59%, SD = 0.35), energy metabolism (mean = 9.89%, SD = 0.27), membrane transport (mean = 8.76%, SD = 0.60) and metabolism of cofactors and vitamins (mean = 8.24%, SD = 0.16). When we analyzed the KO relative abundances across samples, these were clustered by site based on Bray Curtis distances (Figure 2B). We found significant differences (*p*-value < 0.05 and LDA score > 2) between sites at the KO level (Table S2). Specifically, we identified 36 KOs that discriminated between Sob and Sapo sites. Of the 36 KOs, 27 and 9 were enriched in Sob and Sapo, respectively. In Sob, most of the enriched genes were part of functional classes involved in membrane transport (13), cellular communication (5), biosynthesis of secondary metabolites (2) and antimicrobial drug resistance (1). In Sapo, enriched genes were associated with the cell cycle (1), lipid metabolism (1) and xenobiotic degradation metabolism (2). In addition to differences found between sites, there is also individual variation among samples within each site as shown in Figure 2B.

### Genes involved in bacterial communication, transport and defense: unique and shared functional traits between sites

Based on assembled contigs, we specifically decided to explore genes from five functional classes associated with bacterial communication, molecular transport and defense mechanisms: biosynthesis of secondary metabolites (BSM), metabolism of terpenoids and polyketides (MTP), membrane transport (MT), cellular communication (CC) and antimicrobial drug resistance (ADR).

**Figure 3:**
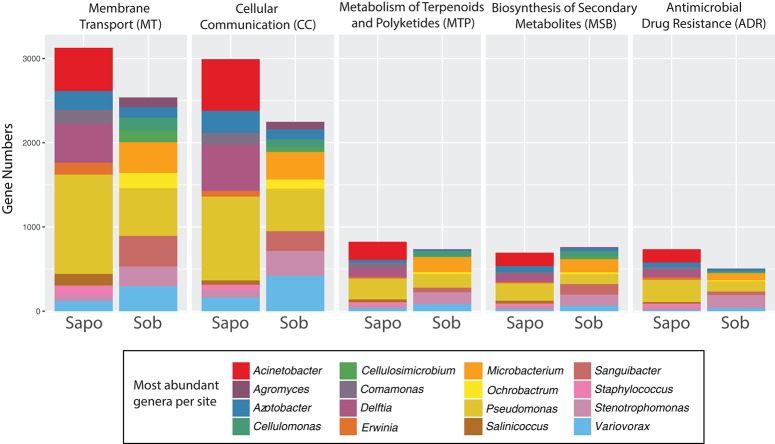
Contig level analysis. Gene abundance stacked graphs of five functional categories from KEGG that are involved in bacterial communication and bacterial-host-pathogen interactions. Each category represents the gene abundance for Sapo (*N* = 3) and Sob (*N* = 3) sites.

Skin metagenomes from both sites had genes for the five functional classes tested, but these genes were associated with different taxonomic groups depending on the site. As seen in Figure 3, most of the genes within each category belonged to *Pseudomonas, Delftia, Azotobacter*, and *Acinetobacter* in Sapo and *Pseudomonas, Variovorax, Sanguibacter, Stenotrophomonas*, and *Microbacterium* in Sob. However, no significant differences in the number of genes were found between Sapo and Sob sites for these functional categories (LefSe *p*-values > 0.05).

We further analyzed the gene abundance for all pathways within each functional category (55 in total). In the case of the BSM class, 16 out of 25 pathways described in KEGG were present in the skin metagenomes. The most abundant were the biosynthesis of monobactam, prodigiosin, streptomycin and novobiocin (Figure S1). In the case of the MTP class, 16 out of 21 pathways described in KEGG were present in the skin metagenomes. The most abundant were terpenoid backbone biosynthesis, geraniol degradation, polyketide sugar unit biosynthesis and biosynthesis of siderophore group nonribosomal peptides (Figure S2). In the case of classes MT, CC and ADR all pathways were present (Figure S3). In the case of the MT class, and as part of the bacteria secretion system pathway, we found almost the complete Type II and Type VI secretion systems, which are involved in secreting molecules (including toxins) to the external environment (Green and Mecsas, 2016). Also, we identified several complete ABC transporters from all the different types of prokaryotic transporters according to KEGG. Within the CC pathways, several genes involved in biofilm formation were identified, as well as quorum sensing genes that had previously been found in Gammaproteobacteria and Bacilli classes. In the case of ADR pathways, we identified most of the genes involved in beta-lactam and vancomycin resistance. In addition, several genes involved in antimicrobial peptide (AMP) resistance were found including the two-component system *PhoQ-PhoP* which has been identified as an important component in pathogenic and symbiotic bacteria to adapt to host environments (Clayton et al., 2017).

**Figure 4:**
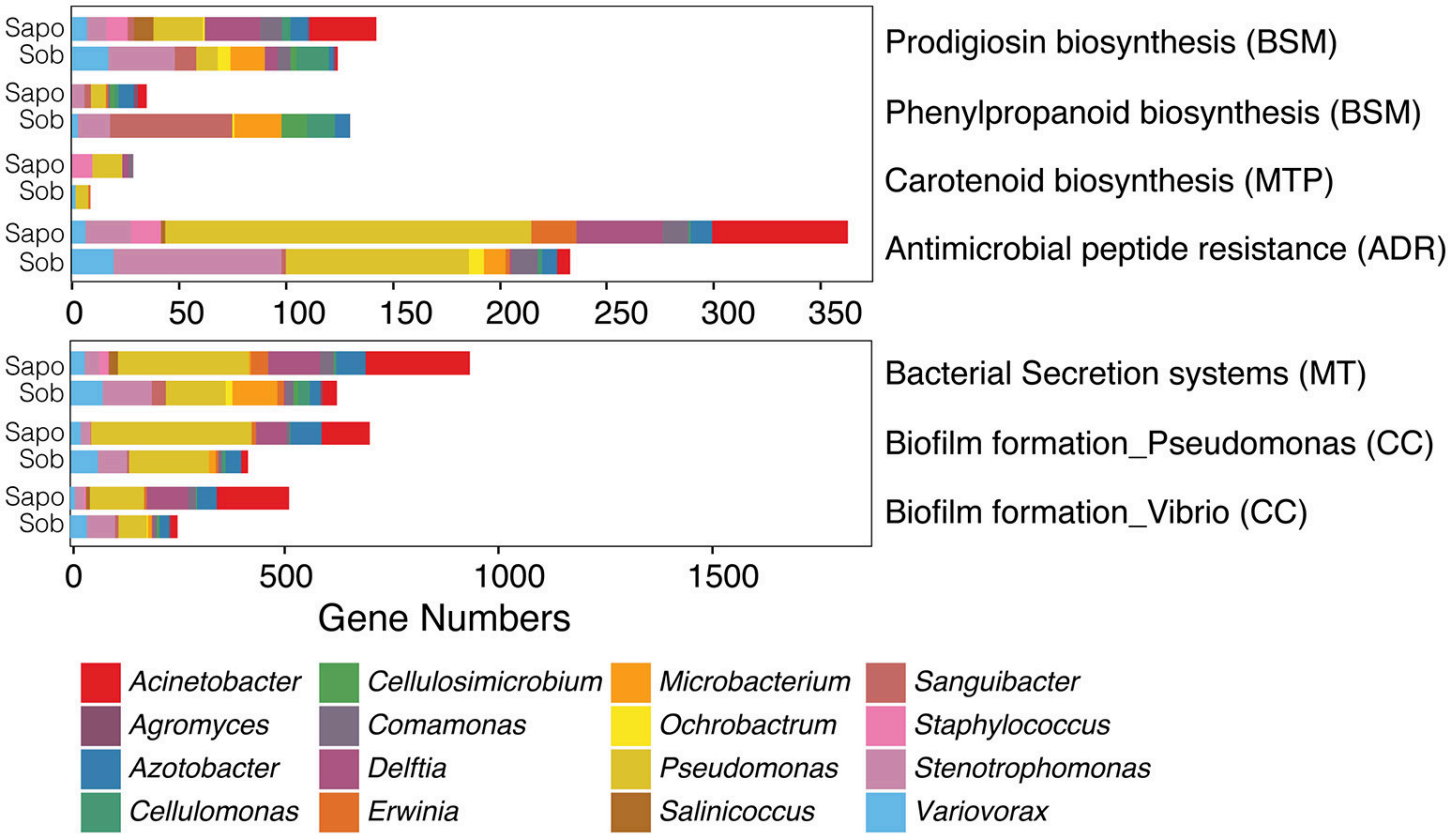
Contig level analysis. Gene abundance plots of significant pathways enriched in Sob or Sapo sites within five functional classes from KEGG involved in bacterial communication and bacterial-host-pathogen interactions. Each bar represents the gene abundances for Sapo (*N* = 3) and Sob (*N* = 3) sites. Colors indicate the taxonomic assignment.

Of all pathways from the five classes, seven were significantly different between sites (LefSe analysis: LDA score > 2 and *p*-value < 0.05) (Figure 4). We found a significant enrichment of phenylpropanoid biosynthesis genes (BSM class) in Sob in comparison to Sapo. Genes from this biosynthetic pathway were enriched in most of the abundant genera in Sob (*Cellulomonas, Cellulosimicrobium, Sanguibacter*, and *Microbacterium*), which were not as abundant in Sapo, and all are from the Actinobacteria class. We found a significant enrichment of prodigiosin biosynthesis genes in Sapo. Genes from this biosynthetic pathway were enriched in most of the abundant genera in Sapo (*Pseudomonas, Agromyces*, and *Acinetobacter*), which are all from the Gammaproteobacteria class. In addition, genes from biofilm formation, AMP resistance, bacterial secretion systems and carotenoid biosynthesis were significantly enriched in Sapo (Figure 4).

We obtained gene co-occurrence networks for each site based on the relative abundance of genes from the same five functional classes (BSM, MTP, MT, CC, and ADR) (Figure S4). Both networks had a similar number of nodes (KOs): 298 nodes in Sapo and 280 nodes in Sob (Table S3). Interestingly, the number of significant Spearman correlations in the Sob network was twofold greater than in the Sapo network (Figure S4) and included an equivalent amount of negative (mutual exclusions) and positive ones (co-occurrences). Accordingly, the clustering coefficient and network centralization were higher in Sob (Table S3). Moreover, the 229 nodes (KOs) shared between the Sob and Sapo networks had strikingly distinct degree values (number of connections per node) (Figure S5). Thus, we did not find a significant correlation between the degree values of the genes shared in the two networks (τ = 0.0239, *p*-value = 0.5987), indicating that connections between nodes are not maintained across sites.

### Anti-Bd secondary metabolite pathways are present in *C. fitzingeri* skin metagenomes

We looked for genes involved in the production of metabolites that have anti-Bd properties such as violacein (Brucker et al., 2008b), indole-3-carboxaldehyde (I3C) (Brucker et al., 2008b), 2,4-diacetylphloroglucinol (2,4-DAPG) (Brucker et al., 2008a), indole-3-ethanol (tryptophol) (Loudon et al., 2014b) and prodigiosin (Woodhams et al., 2017). We identified 16 genes from pathways involved in the production of anti-Bd metabolites (Table 2).

**Table 2:** Genes involved in the production of anti-Bd metabolites that are present on frog skin metagenomes.

**KEGG class**	**KEGG pathway**	**KO**	**Gene description**	**Anti-Bd metabolite**
BSM	Prodigiosin biosynthesis	K00059	fabG; 3-oxoacyl-[acyl-carrier protein] reductase	Prodigiosin
BSM	Prodigiosin biosynthesis	K00208	fabI; enoyl-[acyl-carrier protein] reductase I	Prodigiosin
BSM	Prodigiosin biosynthesis	K00645	fabD; [acyl-carrier-protein] S-malonyltransferase	Prodigiosin
MTP	Terpenoid backbone biosynthesis	K01641	hydroxymethylglutaryl-CoA synthase	2,4 DAPG
MTP	Geraniol degradation	K00022	HADH; 3-hydroxyacyl-CoA dehydrogenase	I3C and tryptophol
MTP	Insect hormone biosynthesis	K00128	ALDH; aldehyde dehydrogenase (NAD+)	I3C and tryptophol
AAM	Tryptophan metabolism	K00164	2-oxoglutarate dehydrogenase E1 component	I3C and tryptophol
BSM	Isoquinoline alkaloid biosynthesis	K00274	MAO, aofH; monoamine oxidase	I3C and tryptophol
AAM	Tryptophan metabolism	K00452	3-hydroxyanthranilate 3,4-dioxygenase	I3C and tryptophol
AAM	Tryptophan metabolism	K00453	tryptophan 2,3-dioxygenase	I3C and tryptophol
AAM	Tryptophan metabolism	K00466	tryptophan 2-monooxygenase	I3C and tryptophol
AAM	Tryptophan metabolism	K01432	arylformamidase	I3C and tryptophol
AAM	Tryptophan metabolism	K01556	kynureninase	I3C and tryptophol
MTP	Limonene and pinene degradation	K01692	paaF, echA; enoyl-CoA hydratase	I3C and tryptophol
AAM	Tryptophan metabolism	K10217	aminomuconate-semialdehyde/2-hydroxymuconate-6-semialdehyde dehydrogenase	I3C and tryptophol
AAM	Tryptophan metabolism	K14338	cypD_E, CYP102A2_3	I3C and tryptophol

*The first column indicates broad functional classes: BSM, Biosynthesis of secondary metabolites; MTP, Metabolism of terpenoids and polyketides; AAM, Amino acid metabolism*.

Specifically, we identified three genes from the prodigiosin biosynthesis pathway (BSM) that were present in both sites and significantly enriched in Sapo (Figure 4). One or more genes from this pathway were present in all the abundant bacterial taxa from both sites except for *Erwinia*, which was only abundant in Sapo. We also identified the coding gene of the enzyme responsible for producing 2,4 DAPG from the terpenoid backbone biosynthesis pathway (MTP) in both sites (K01641). This gene was found only in *Staphyloccocus* (abundant in Sapo) and *Microbacterium* (abundant in Sob). In addition, we identified 12 genes that are part of the tryptophan metabolism from which I3C and tryptophol are produced. Specifically, we detected the enzyme that produces indole-3-acetic acid (K00128), from which I3C can be produced (Stutz, 1958). Most of these genes, including K00128, were present in all the abundant bacterial taxa from both sites with a few exceptions (see Table S4).

## Discussion

In this study, we described the taxonomic and functional diversity of *C. fitzingeri* skin metagenomes, and we compared these microbiomes between two populations, one being from a Bd-endemic site (Sob) and the other from a Bd-naïve site (Sapo). To our knowledge, this is the first study describing the functional diversity of skin microbiomes on adult amphibians using shotgun metagenomics. First, we discuss genes and pathways found in both populations that may be required for bacterial interactions and bacterial-host-pathogen interactions. Then, we discuss differences between populations in genes and pathways. We used two approaches (read-level and contig-level) that showed consistent results, although contig-level analyses allowed us to better describe taxonomic and functional features present on *C. fitzingeri* skin metagenomes.

We focused on the analysis of five main functional classes that could inform us on the capacity of these bacterial communities to communicate with each other, to interact with the host, and to interact with pathogens like Bd: membrane transport (MT), cell communication (CC), biosynthesis of secondary metabolites (BSM), metabolism of terpenoid and polyketides (MTP) and antimicrobial drug resistance class (ADR). Many of the pathways present in these five functional classes are considered essential for bacteria to communicate with each other and to respond to stimuli from the environment. Examples of these pathways are ABC transporters (MT) (Davidson et al., 2008), quorum sensing (CC) (Rutherford and Bassler, 2012), biofilm formation (CC) (Moons et al., 2009), and secondary metabolite production (BSM and MTP) (Flórez et al., 2015). Here, we found that these essential pathways were prominent in *C. fitzingeri* skin metagenomes independent of bacterial community structure. Based on these results, it appears that many different bacterial taxa can provide the same functions within these communities, suggesting that functional redundancy may be one of the properties present in these symbiotic communities (Foster et al., 2017). Thus, differences in community structure that have been identified among populations and sites in previous studies, may not always mean these communities differ in function (Belden et al., 2015).

In the case of symbiotic bacteria in other hosts, some genes and pathways within these functional classes are known to be important for bacteria to adapt to the host environment, such as the mammalian gut (Frese et al., 2013). One example is the bacterial secretion system pathways (MT), which were initially described in pathogenic bacteria, but have been recently described in symbiotic bacteria (Green and Mecsas, 2016). The presence of almost the complete set of genes for secretion systems II and VI in *C. fitzingeri* metagenomes suggest that skin bacteria export molecules (perhaps toxins) through these mechanisms and may in turn exert an effect on their host. In addition to the secretion system, other pathways can be important for bacteria to survive on the host; such is the case of the antimicrobial peptide (AMP) production pathways within the ADR class. In *C. fitzingeri* metagenomes, we found genes from the AMP production pathway. Symbiotic skin bacteria would be expected to evolve mechanisms to avoid the effects of AMPs to colonize the skin. Specifically, the presence of genes within the AMP resistance pathway suggests that skin microbiomes can adapt to the host environment in part through this mechanism. Many amphibian species produce a vast array of AMPs (Conlon, 2011) but so far, no information on these defense molecules has been published for *C. fitzingeri*.

Skin metagenomes of *C. fitzingeri* also have a broad range of secondary metabolite pathways (BSM, MTP and ADR classes). These pathways include the biosynthesis of antibiotics, toxins and aromatic compounds, as well as antibiotic resistance pathways, which may be involved in bacterial competition within the community but could also play a role in protecting the host against pathogens. Indeed, it has been proposed that defense of the host arises as a byproduct of microbial competition (Scheuring and Yu, 2012). One example is the microbial symbionts from sponges and corals which produce terpenoids and polyketides (MTP) and play a protective role (Flórez et al., 2015). In *C. fitzingeri* metagenomes, we identified genes from the MTP class involved in terpenoid and polyketide biosynthetic pathways, antibiotic biosynthesis pathways like ansamycins and vancomycin from the MTP class and antibiotic resistance pathways within the ADR class.

The MTP class also includes pathways involved in the degradation of several compounds produced mainly by plants such as geraniol, pinene and limonene. These pathways have been previously described in other bacterial genera like *Pseudomonas* and *Rhodococcus* (Marmulla and Harder, 2014), which can use these toxic compounds as carbon sources. We found these pathways present in *C. fitzingeri* metagenomes, which is expected considering that frogs are exposed to many of the same or similar carbon sources in their leaf-litter habitat. Also, terpenoid degradation pathways have been found in other bacterial symbionts (Marmulla and Harder, 2014).

We expected to find genes for the production of anti-Bd metabolites, since *C. fitzingeri* is apparently resistant or tolerant to Bd infection in the wild. We identified some of the genes involved in the production of prodigiosin, I3C, tryptophol and 2,4 DAPG in both sites (Sapo and Sob). However, proving whether these metabolites are produced on *C. fitzingeri* skin communities would require additional studies that test the presence of their respective transcripts or the metabolites themselves *in vivo*.

In this study, we identified clear differences in bacterial community structure between sites that were consistent with previous studies using 16S rRNA gene amplicon sequencing (Rebollar et al., 2016). Some members of these skin communities were shared between sites, but some genera were clearly enriched in either Sapo or Sob. Based on the results obtained here, we consider that the genetic/functional differences between sites may be explained by the unique bacterial genera found in each of the populations that we studied. We expect bacterial communities in both sites to provide a defensive function since potential pathogens are ubiquitous in nature; however, we also expected increased selection for a defensive function in Sob since this is considered a Bd-endemic site (Rebollar et al., 2014).

One clear example of the differences between sites in terms of function and taxonomic composition is the enrichment of genes from the phenylpropanoid biosynthesis pathway at the Sob site (Figure 4). Phenylpropanoids are a diverse family of metabolites that are common plant natural products and play several roles, including resistance to pests in plants (reviewed in Vogt, 2010). In this study, most of the bacteria harboring genes from this biosynthetic pathway in Sob were *Sanguibacter, Microbacterium, Cellulomonas*, and *Cellulosimicrobium*, which are all Actinobacteria. The Phenylpropanoid biosynthesis pathway has been previously found in Actinobacteria (Moore et al., 2002), and several bioactive molecules from this family have been identified using metabolomics (Wu et al., 2016). In addition, members of the Actinobacteria class also produce a wide range of antibiotics, and have been identified as symbionts that play a crucial role for the protection of several animal hosts (mainly insects) against pathogens (Flórez et al., 2015). Considering that Sob is a Bd-endemic site, we hypothesize that the enrichment of Actinobacteria in Sob provides *C. fitzingeri* with protection against the pathogen Bd. Further analyses would be necessary to determine if the presence of Actinobacteria in *C. fitzingeri* skin indeed plays a role in protecting the host against Bd and which molecular mechanisms are involved. We also identified six metabolic pathways that were enriched in Sapo from the CC, ADR, MT and BSM functional classes (Figure 4) that were mainly explained by genes associated to *Acinetobacter, Pseudomonas*, and *Delftia*.

The functional traits enriched in either Sapo or Sob likely reflect distinct interactions within the members of the skin microbiome and potentially different ways to interact with the host. Moreover, the differences found in co-occurrence networks between sites (mainly on the number of connection that nodes have) may be caused by the presence of distinct taxonomic groups that harbor unique genetic repertoires. In particular, the functional repertoire of Actinobacteria in Sob and Gammaproteobacteria in Sapo may cause distinct degree values of the KOs within each network.

We suggest that shotgun metagenomics is a promising tool that could allow a deeper understanding of the functions present in amphibian skin microbiomes. This approach could be used not only in the field but also in experimental settings since it could unveil functional changes in time-series or bacterial manipulation experiments (Davis et al., 2017). In the case of the *C. fitzingeri* skin microbiome, we have been able to describe important pathways involved in bacterial communication, as well as genes involved in potential bacterial-host-pathogen interactions. However, an important caveat of this study is the sample size (3 microbiomes per site) which may influence our results and may not allow us to detect other significant differences between sites. Thus, in the future, we strongly suggest increasing the sample size to fully describe the functional diversity present in amphibian skin microbiomes.

## Ethics statement

Scientific collection permits were provided by the Panamanian authorities (Autoridad Nacional del Ambiente): permits SE/A-47-12, SEX/A-65- 12, SEX/A-77-12 and SEX/A-89-12. Animal care protocols were approved by the Smithsonian Tropical Research Institute's Animal Care Committee: protocol 2011-1110- 2014 and by Virginia Tech's Animal Care Committee: protocol 11-105- BIOL.

## Author contributions

ER, RH, and LB: contributed to the original idea; ER, RH, LB, RJ, JW, and MH: contributed to the design of the research; MH and DM: carried out the fieldwork; ER, JW, MH, and DM: contributed with laboratory analyses; ER, RJ, AG-P, CN, and AE: performed all data analyses; ER, AG-P, CN, AE, EB, and RH: contributed to analyses interpretation; ER: wrote the manuscript and all authors provided critical feedback.

### Conflict of interest statement

The authors declare that the research was conducted in the absence of any commercial or financial relationships that could be construed as a potential conflict of interest.
