# Supplementary material for: The Skin Microbiome of the Neotropical Frog Craugastor fitzingeri: Inferring Potential Bacterial-Host-Pathogen Interactions From Metagenomic Data
Source: Front Microbiol. 2018 Mar 20;9:466. doi: 10.3389/fmicb.2018.00466 (PMC5869913; doi:10.3389/fmicb.2018.00466)
Supplement: Supplementary file 1 [file Table1.docx]

**Table S1.** Description of frog skin shotgun metagenomes, including location and Bd infection loads, number of reads obtained per sample before and after filtering, and number (proportion) of annotated reads.

| **Sample** | **Location**  **(Province)** | **Bd infection loads (zeq)** | **Total number of reads** | **Number of reads after filtering out frog-associated reads** | **Number of reads with KO annotations** |
| --- | --- | --- | --- | --- | --- |
| Sob01 | Soberanía National Park (Panamá) | 0 | 99,863,466 | 97,701,454 | 31,264,532 (32%) |
| Sob02 | Soberanía National Park (Panamá) | 188 | 84,427,294 | 82,623,892 | 33,956,588 (41%) |
| Sob03 | Soberanía National Park (Panamá) | 22 | 56,402,570 | 56,375,008 | 26,494,447 (47%) |
| Sapo01 | Serranía del Sapo (Darién) | 0 | 75,053,356 | 72,204,150 | 18,320,318 (25.4%) |
| Sapo02 | Serranía del Sapo (Darién) | 0 | 98,118,154 | 96,627,348 | 28,659,036 (29.6%) |
| Sapo03 | Serranía del Sapo (Darién) | 0 | 124,761,208 | 124,643,032 | 54,066,648 (43.4%) |
| TOTAL |  |  | 538,626,048 | 530,174,884  (98.43% of the total number of reads) | 192,761,569 (36.36% of the filtered reads) |
