# Supplementary material for: The Skin Microbiome of the Neotropical Frog Craugastor fitzingeri: Inferring Potential Bacterial-Host-Pathogen Interactions From Metagenomic Data
Source: Front Microbiol. 2018 Mar 20;9:466. doi: 10.3389/fmicb.2018.00466 (PMC5869913; doi:10.3389/fmicb.2018.00466)
Supplement: Supplementary file 3 [file Table3.docx]

**Table S3.** Indices of the networks for Sapo and Sob sites. Nodes correspond to genes (KOs) and edges correspond to significant Spearman correlations (p-value < 0.001 and -0.8 < R > 0.8).

| **Network Index** | **Sapo** | **Sob** |
| --- | --- | --- |
| **Number of nodes** | 298 | 280 |
| **Number of edges** | 2405 | 4632 |
| **Network Centralization** | 0.264 | 0.476 |
| **Clustering Coefficient** | 0.238 | 0.337 |
| **Betweenness Centrality** | 0.006 | 0.004 |
| **Closeness Centrality** | 0.372 | 0.464 |
| **Power Law of node degree, R2** | 0.775 | 0.451 |
