# Supplementary material for: The Skin Microbiome of the Neotropical Frog Craugastor fitzingeri: Inferring Potential Bacterial-Host-Pathogen Interactions From Metagenomic Data
Source: Front Microbiol. 2018 Mar 20;9:466. doi: 10.3389/fmicb.2018.00466 (PMC5869913; doi:10.3389/fmicb.2018.00466)
Supplement: Supplementary file 5 [file DataSheet1.docx]

Supplementary Material

**The skin microbiome of the Neotropical frog *Craugastor fitzingeri*: Inferring potential bacterial-host-pathogen interactions from metagenomic data**

Eria A. Rebollar^1,$*^, Ana Gutiérrez-Preciado^2^, Cecilia Noecker^3^, Alexander Eng^3^, Myra C. Hughey^4^, Daniel Medina^4^, Jenifer B. Walke^4,&^, Elhanan Borenstein^3,5,6^, Roderick V. Jensen^4^, Lisa K. Belden^4,7^, Reid N. Harris^1,8^.

^1^Department of Biology, James Madison University, Harrisonburg, VA, USA.

^2^Unité d'Ecologie, Systématique et Evolution, Université Paris-Sud, Paris, France.

^3^Department of Genome Sciences, University of Washington, Seattle, WA,USA.

^4^Department of Biological Sciences, Virginia Tech, Blacksburg, VA, USA

^5^Department of Computer Science and Engineering, University of Washington, Seattle, WA, USA.

^6^Santa Fe Institute, Santa Fe, NM, USA.

^7^ Smithsonian Tropical Research Institution, Panama City, Panama.

^8^Amphibian Survival Alliance, London, United Kingdom.

^$^ Present address: Departamento de Ecología Evolutiva, Instituto de Ecología, Universidad Nacional Autónoma de México, Mexico City, Mexico

^&^ Present address: Department of Biology, Eastern Washington University, Cheney, WA, USA

* Correspondence:
Eria A. Rebollar
[ea.rebollar@gmail.com](mailto:ea.rebollar@gmail.com)

**SUPPLEMENTARY FILES**

1. **Supplementary Methods**
2. **Supplementary References**
3. **Supplementary Methods**

**Network Reconstruction**

From a list of 2,391 genes (KOs) from the five broad functional classes mentioned in the previous section and the 55 individual pathways therein, one matrix was built for each of the sampling sites (2 matrices in total) containing the gene abundance per frog as observed in the metagenomes. Only abundant genes were retained for further co-occurrence network reconstruction: genes whose abundance was less than 5% of the abundance of the most abundant gene in each site were discarded. A total of 280 KOs remained for Sob, and 298 for Sapo. Co-occurrences of these KOs were calculated with SparCC (Friedman & Alm, 2012) for each frog metagenome using the un-normalized gene abundances. Ten iterations were used to estimate the median correlation of each pair and the statistical significance of the correlations was calculated by bootstrapping with 500 iterations. Correlated pairs were retained if R>0.8 or R<-0.8 and P<0.001. Networks were reconstructed using *ad hoc* scripts in R (R Core Team 2015) with the aid of the igraph package (<http://igraph.org/>). Networks were further visualized with Cytoscape (Christmas et al., 2005). To determine whether degree values of the shared KOs (nodes) on Sob and Sapo networks were correlated, we used Kendall rank correlation coefficient in R (R Core Team 2015).

1. **Supplementary References**

Christmas R., Avila-Campillo I., Bolouri H., Schwikowski B., Anderson M., Kelley R., et al. (2005). Cytoscape: A Software Environment for Integrated Models of Biomolecular Interaction Networks. *AACR Education Book* 2005:12–16.

Friedman, J., and Alm, E. J. (2012). Inferring Correlation Networks from Genomic Survey Data. *PLoS Comput. Biol.* 8, 1–11. doi:10.1371/journal.pcbi.1002687.

R Core Team (2015). R: A language and environment for statistical computing. R Foundation for Statistical Computing, Vienna, Austria. URL http://www.R-project.org/
