# Supplementary figures and images for: The Skin Microbiome of the Neotropical Frog Craugastor fitzingeri: Inferring Potential Bacterial-Host-Pathogen Interactions From Metagenomic Data
Source: Front Microbiol. 2018 Mar 20;9:466. doi: 10.3389/fmicb.2018.00466 (PMC5869913; doi:10.3389/fmicb.2018.00466)

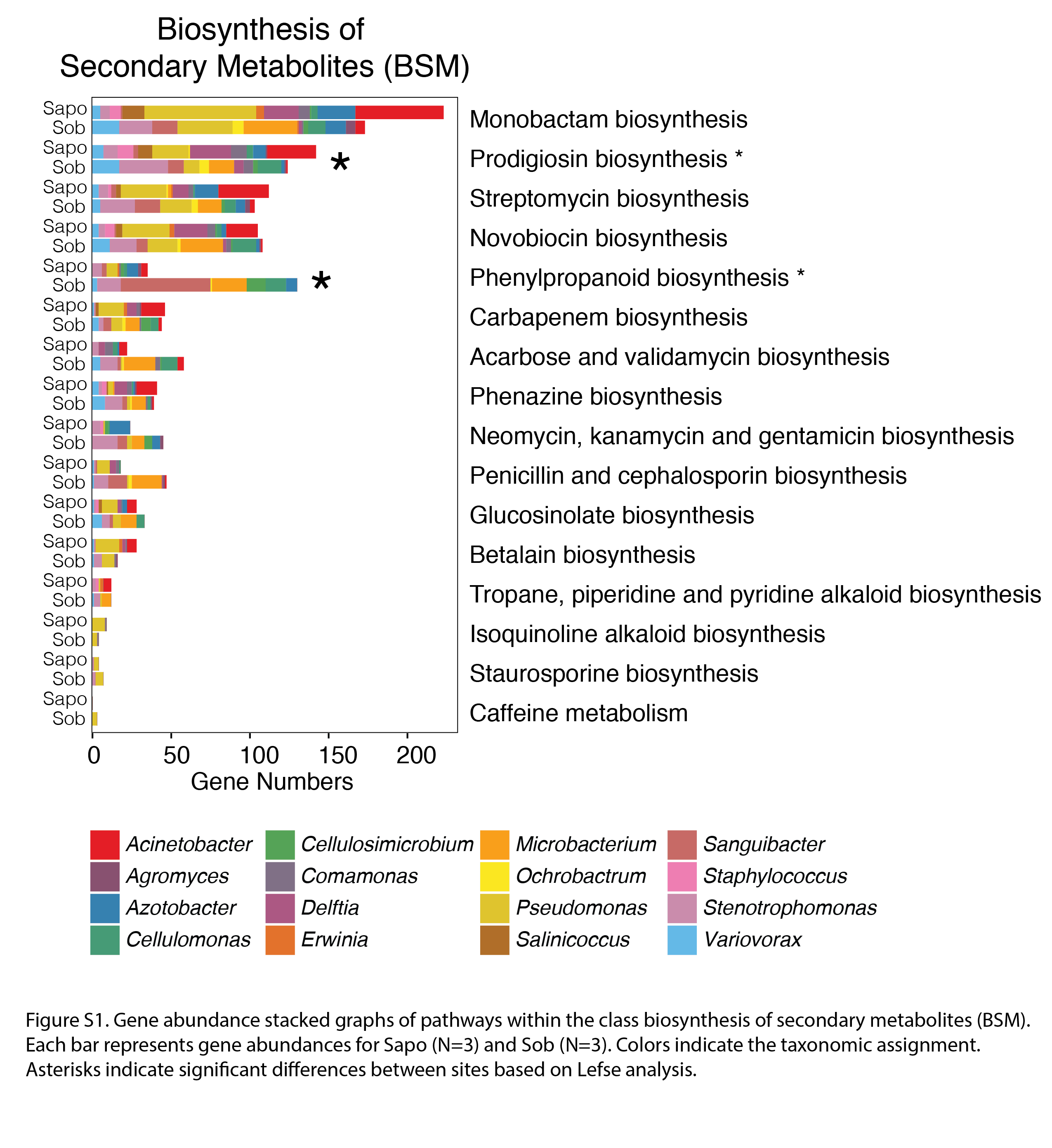

Supplement: Supplementary file 6 [file Image1.png]

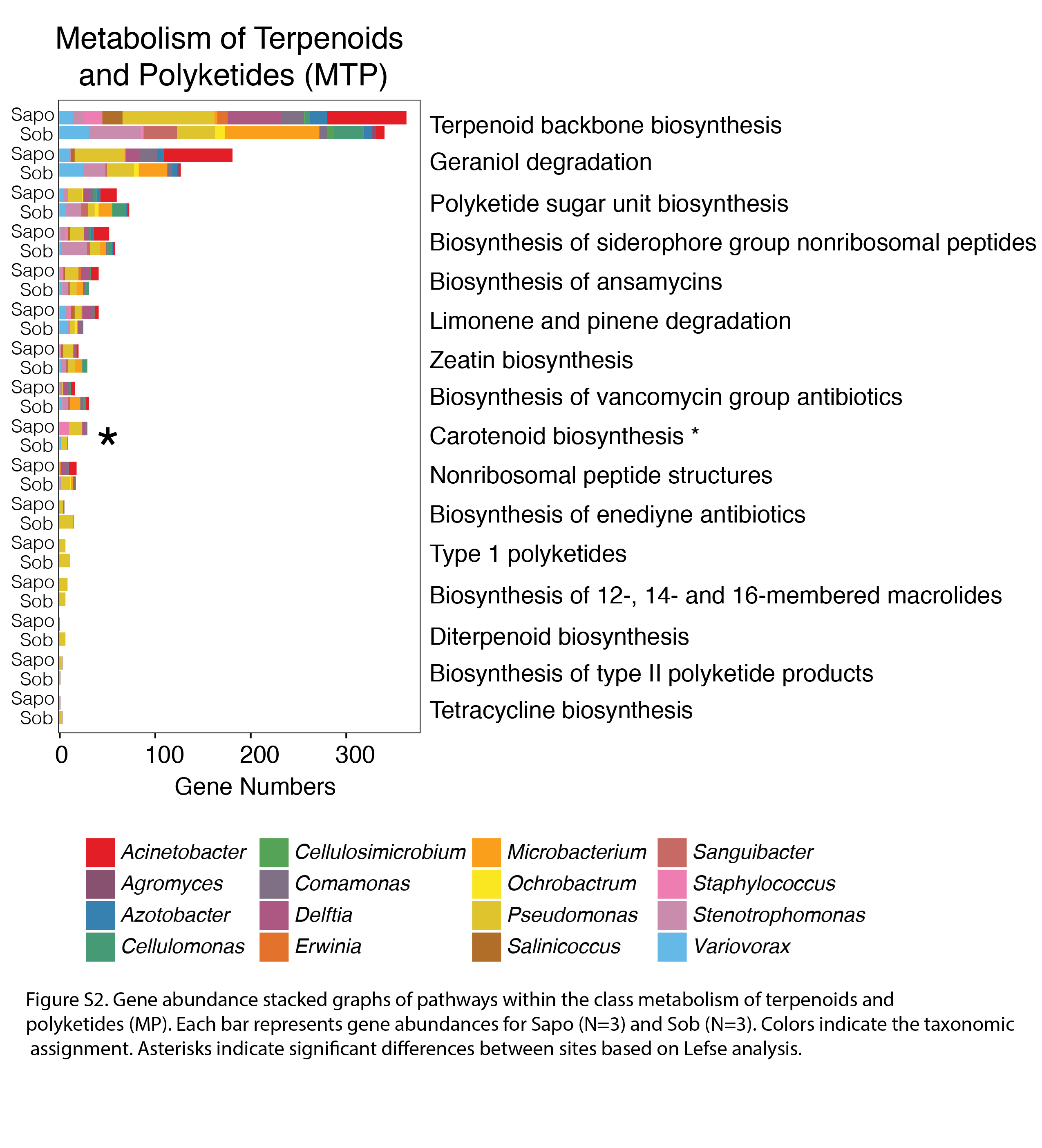

Supplement: Supplementary file 7 [file Image2.png]

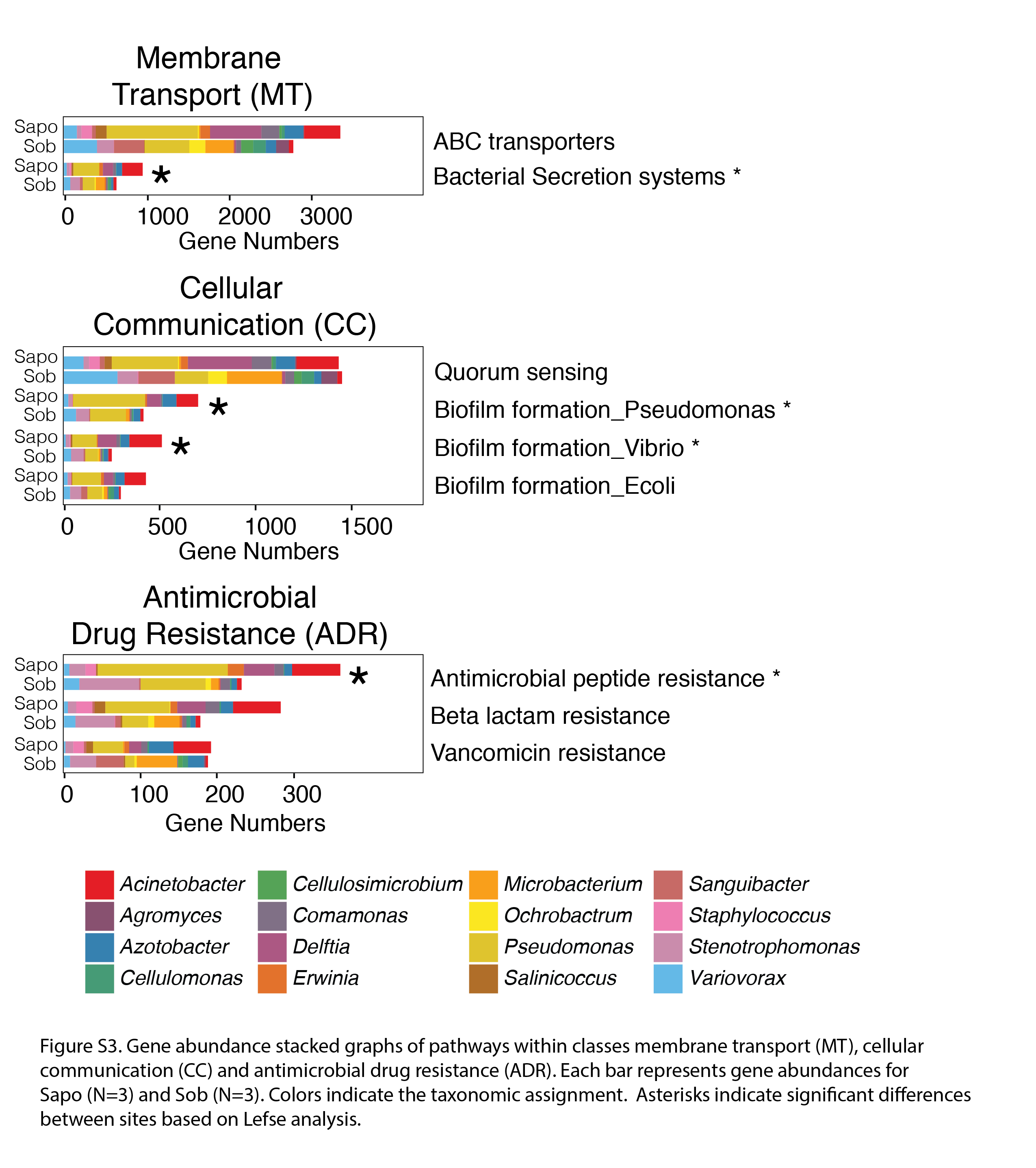

Supplement: Supplementary file 8 [file Image3.png]

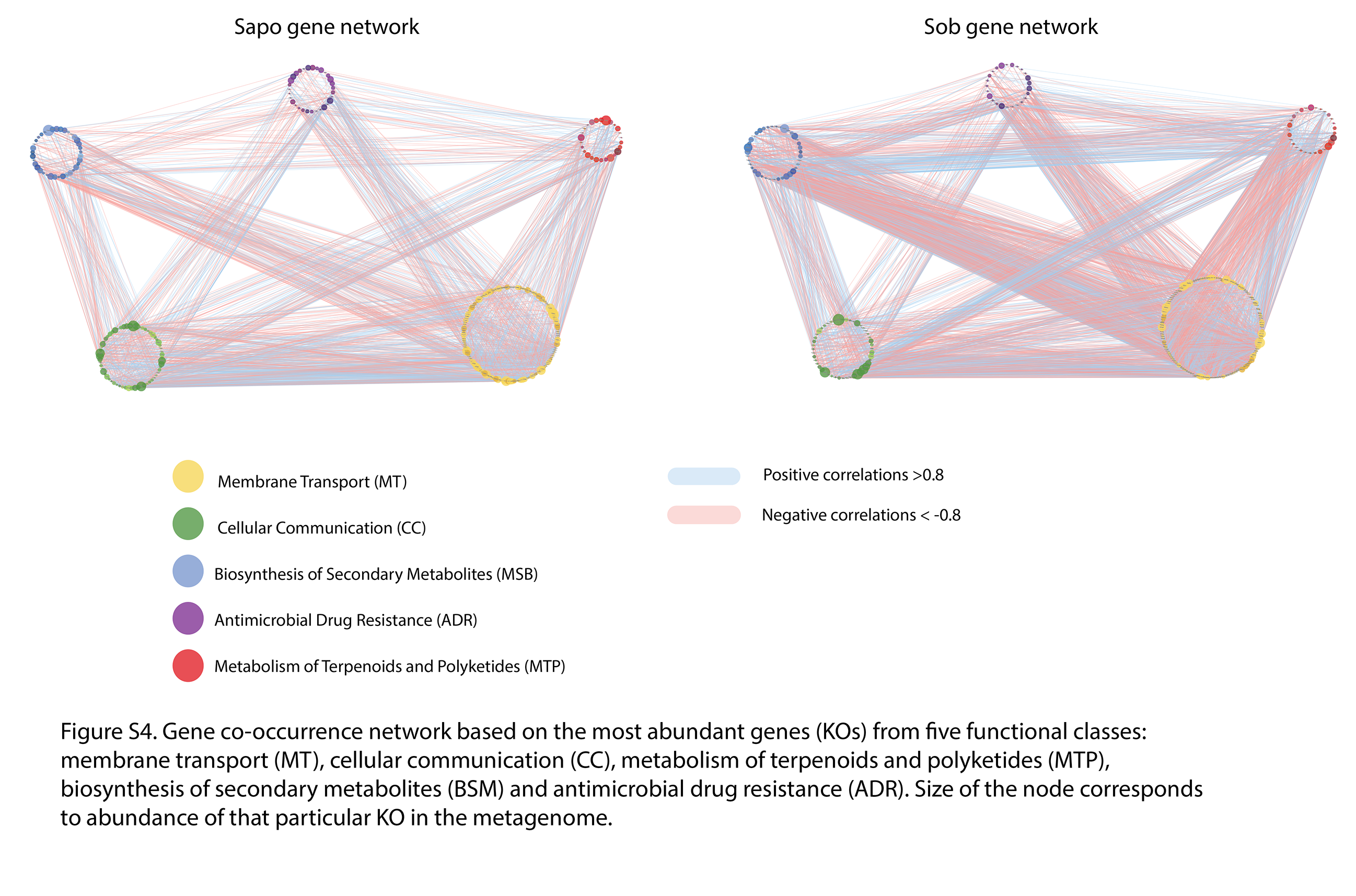

Supplement: Supplementary file 9 [file Image4.png]

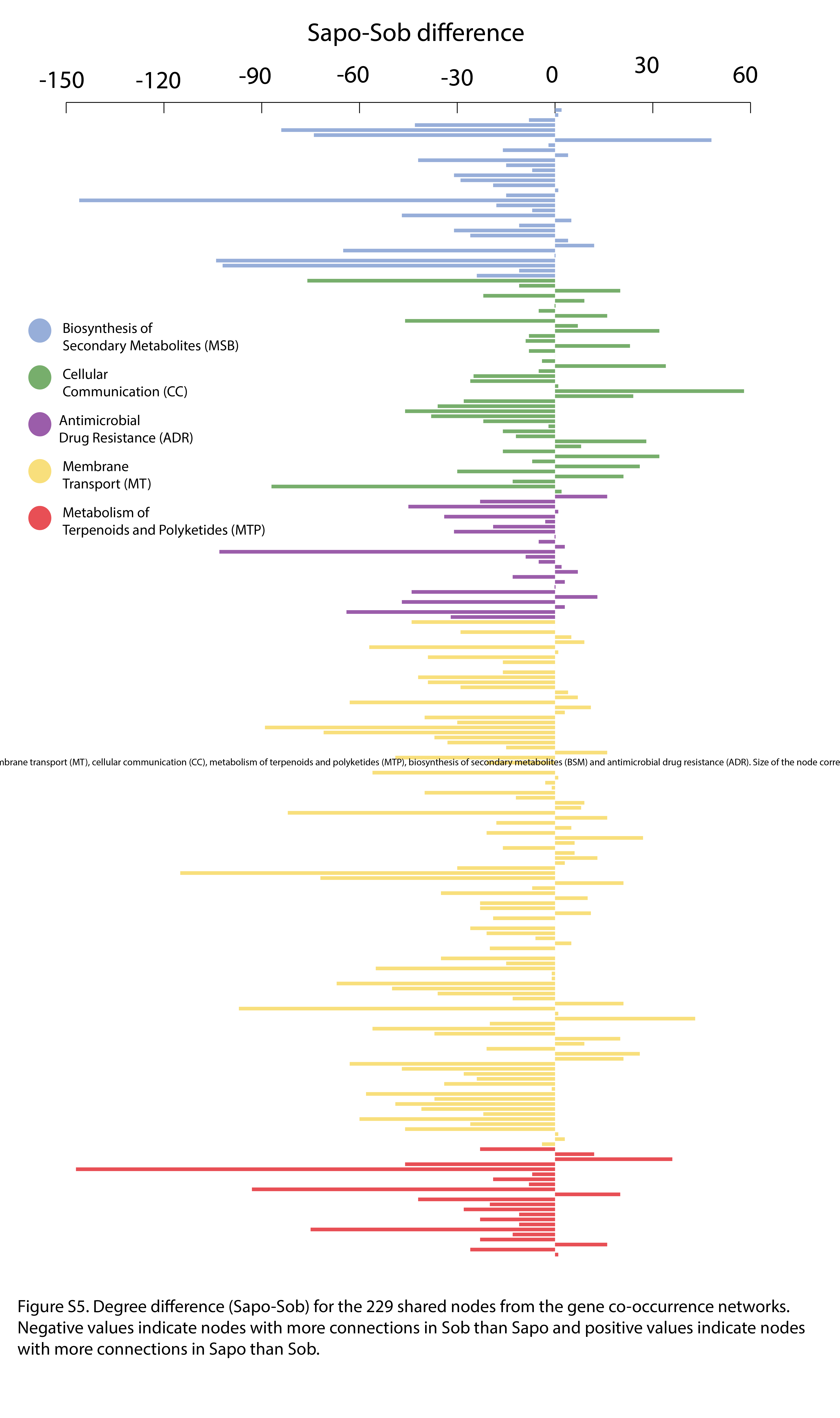

Supplement: Supplementary file 10 [file Image5.png]
